# Influence of Cation Substitution on Cycling Stability and Fe-Cation Migration in Li_3_Fe_3–*x*_M_*x*_Te_2_O_12_ (M = Al, In) Cathode Materials

**DOI:** 10.1021/acs.inorgchem.3c03929

**Published:** 2024-01-04

**Authors:** Xabier Martínez de Irujo Labalde, Man Yi Lee, Heather Grievson, Josie-May Mortimer, Samuel G. Booth, Emmanuelle Suard, Serena A. Cussen, Michael A. Hayward

**Affiliations:** † Department of Chemistry, Inorganic Chemistry Laboratory, University of Oxford, South Parks Road, Oxford OX1 3QR, U.K.; ‡ The Faraday Institution, Quad One, Harwell Campus, Didcot OX11 0RA, U.K.; § Department of Materials Science and Engineering, Sir Robert Hadfield Building, University of Sheffield, Sheffield S1 3JD, U.K.; ∥ Institut Laue-Langevin, 71 Avenue des Martyrs, Grenoble 38000, France

## Abstract

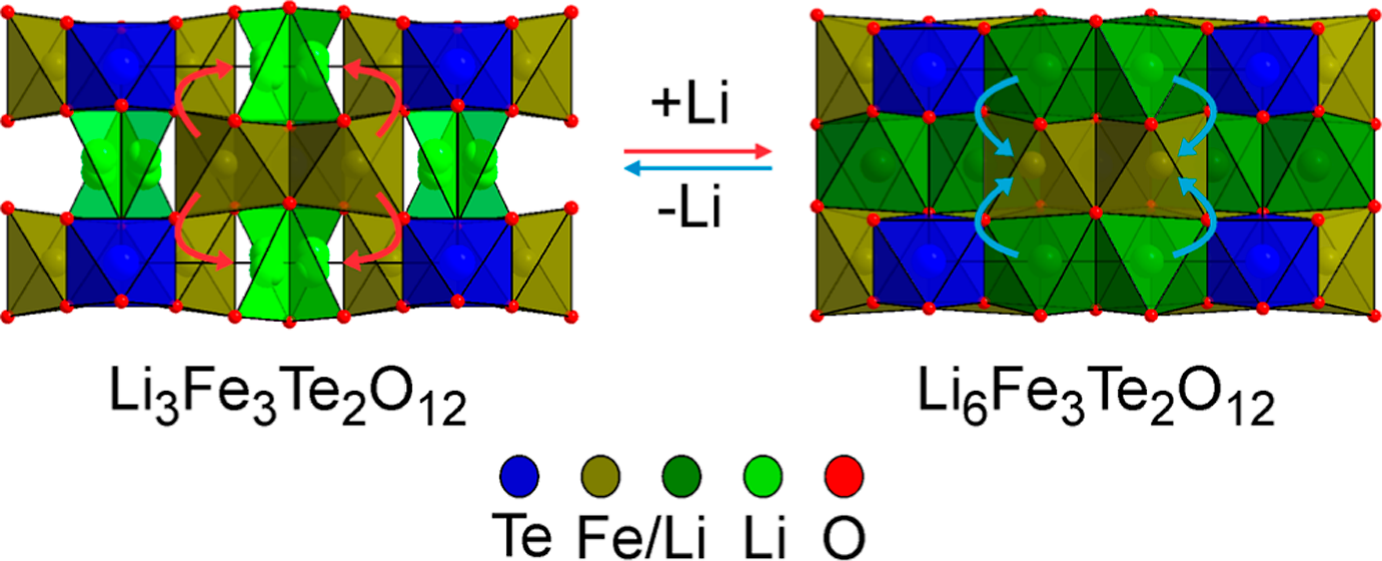

Li_3_Fe_3_Te_2_O_12_ adopts a crystal structure, described in space group *Pnnm*, related to that of LiSbO_3_, in which Te^6+^, Fe^3+^, and Li^+^ cations reside in a partially ordered configuration within an hcp array of oxide ions. Chemical or electrochemical insertion of lithium is accompanied by a fully reversible migration of some of the Fe cations with an initial capacity of 120 mA h g^–1^ (2.85 Li per formula unit). Long-term cycling stability is limited by the facile reduction of Te^6+^ to elemental Te, which leads to cathode decomposition. Partial substitution of Fe by In suppresses Te^6+^ reduction, such that Li_3_Fe_2_InTe_2_O_12_ shows no sign of this cathode decomposition pathway, even after 100 cycles. In contrast, Al-for-Fe substitution is chemically limited to Li_3_Fe_2.6_Al_0.4_Te_2_O_12_ and appears to have almost no influence on cathode longevity. These features of the Li_3_Fe_3-x_M_*x*_Te_2_O_12_ system are discussed on the basis of a detailed structural analysis performed using neutron and synchrotron X-ray diffraction.

## Abstract 2

Lithium insertion into Li_3_Fe_3_Te_2_O_12_ results in large-scale Fe-cation migration, with repeated electrochemical cycling resulting in cathode decomposition via reduction of Te^6+^ to elemental Te. Substitution of 1/3 of the Fe^3+^ by In^3+^ limits the degree of Fe-cation migration and stabilizes the material with respect to Te^6+^ reduction.

## Introduction

The high energy density of Li-ion batteries has made them the power source of choice in a wide variety of applications.^1−3^ However, if Li-ion batteries are to be widely utilized for applications such as grid-scale storage for renewable power generation or in electric vehicles, their cathode chemistry will need to be modified to replace expensive and toxic elements such as Co or Ni, with cheaper, more earth-abundant alternatives.^4,5^

Cathode materials based on the redox chemistry of iron have been proposed to fulfill this need due to the low cost, high abundance, and low toxicity of iron compounds.^6^ However, there are a number of features of iron chemistry that make the development of high-capacity Fe-based cathode materials challenging. For example, while many stable Fe^4+^ oxides can be prepared,^7,8^ the high potential of the Fe^III^/Fe^IV^ redox couple (while attractive in terms of energy storage) tends to lead to irreversible anion-redox processes and/or oxygen loss when utilized in cathode materials.^9,10^ Furthermore, iron-based cathode materials tend to exhibit large-scale cation migration during charge/discharge cycles.^11,12^ This is because the S = ^5^/_2_, high-spin configurations adopted by Fe^3+^ centers in oxide environments have no strong ligand-field-driven coordination geometry preference, with both octahedral and tetrahedral coordination common. Oxidation of Fe^3+^ centers on cathode charging generates S = 2, Fe^4+^ centers, which have a strong preference for octahedral geometry, and when combined with a large change in ionic radius (Fe^3+^ = 0.645 Å, Fe^4+^ = 0.585 Å),^13^ this provides a large driving force for structural rearrangement during electrochemical cycling. These undesirable features can be seen during the electrochemical cycling of different LiFeO_2_ polymorphs, which are irreversibly converted to a LiFe_5_O_8_ spinel phase on lithium extraction via a combination of oxygen loss and cation migration.^10,14,15^

By utilizing the Fe^II^/Fe^III^ redox couple in place of the Fe^III^/Fe^IV^ couple, problems relating to anion redox can be avoided at the expense of reduced cell potential and energy storage capacity. However, the issue of cation migration and phase instability remains as S = 2, Fe^2+^ centers exhibit a strong octahedral coordination preference and are significantly larger than Fe^3+^ centers (Fe^2+^ = 0.78 Å).^13^

Recently, we have been trying to address the issue of cation migration in Fe-based cathode materials by preparing a series of “model” systems, so we can better study this behavior and thus develop strategies to minimize it. As part of this approach, we have been using post-transition metals such as Sb, In, or Te to stabilize Li–Fe–M–O phases in LiSbO_3_-like structures and have then used cation substitution to suppress Fe-cation migration on lithium insertion. For example, lithium can be readily inserted into LiFe_2_SbO_6_,^16^ but this process is associated with large-scale Fe-cation migration, resulting in a phase, Li_2_Fe_2_SbO_6_, that exhibits cation-order and Fe^2+/3+^ charge-order, which prevents subsequent Li extraction and thus electrochemical cycling of the material. Partial In-for-Fe substitution suppresses this Fe-cation migration and charge order, allowing repeated redox cycling of the material. Here, we describe the preparation of the structurally related phase, Li_3_Fe_3_Te_2_O_12_, and describe the effects of cation substitution on Fe-cation migration observed during redox cycling.

## Experimental Section

### Sample Preparation

Samples of Li_3_Fe_3–*x*_In_*x*_Te_2_O_12_ (*x* = 0, 1) and Li_3_Fe_3–*x*_Al_*x*_Te_2_O_12_ (*x* < 0.4) were synthesized by a ceramic method by grinding together suitable ratios of Fe_2_O_3_ (Alfa Aesar, 99.995%), In_2_O_3_ (Alfa Aesar, 99.995%), Al_2_O_3_ (99.995%), TeO_2_ (Alfa Aesar, 99.999%), and Li_2_CO_3_ (Alfa Aesar, 99.95%) in an agate mortar and pestle. The mixtures were placed in alumina crucibles and heated in air at 600 °C for 12 h. The powders were then reground, pressed into 13 mm pellets, and heated at 775 °C for 12 h in air.

Attempts to intercalate additional lithium within Li_3_Fe_3–*x*_In_*x*_Te_2_O_12_ samples were performed by stirring approximately 2 g of material in 15 mL of a 1.4 M solution of *n*-BuLi in toluene (Sigma-Aldrich) under a nitrogen atmosphere for up to 5 days at room temperature. Intercalation into Li_3_Fe_2.6_Al_0.4_Te_2_O_12_ was performed on a smaller scale with samples of ∼300 mg reacted with 5 mL of 1.4 M *n*-BuLi in toluene. After the reaction, samples were filtered and washed with clean toluene under a nitrogen atmosphere on a Schlenk line. Samples were stored under an inert atmosphere in an argon-filled glovebox.

Chemical reoxidation reactions were carried out by stirring the lithiated material with a large excess of I_2_ in acetonitrile for 4 h at room temperature. Samples were then filtered and washed with clean acetonitrile and then acetone in air.

### Characterization

Reaction progress and initial structural characterization were performed using laboratory powder X-ray diffraction (PXRD) data collected using a PANalytical X’pert diffractometer incorporating an X’celerator position-sensitive detector (monochromatic Cu Kα_1_ radiation). High-resolution synchrotron powder X-ray diffraction (SXRD) data were collected using the I11 instrument at Diamond Light Source Ltd. Diffraction patterns were collected using Si-calibrated X-rays with an approximate wavelength of 0.825 Å from samples sealed in 0.3 mm diameter borosilicate glass capillaries. Neutron powder diffraction (NPD) data were collected using the D2B diffractometer (λ = 1.594 Å) at the ILL neutron source from samples sealed under argon in 8 mm vanadium cans. Rietveld refinement was performed using the TOPAS suite of programs (v6).^17^

X-ray absorption experiments were performed at the B18 beamline of the Diamond Light Source. The measurements were carried out using the Pt-coated branch of the collimating and focusing mirrors, a Si(111) double-crystal monochromator, and a pair of harmonic rejection mirrors. The size of the beam at the sample position was approximately 600 μm × 700 μm. X-ray absorption near-edge spectroscopy data were collected at the Fe K-edge (7112 eV) in transmission mode with ion chambers before and behind the sample filled with appropriate mixtures of inert gases to optimize sensitivity (*I*_0_: 300 mbar of N_2_ and 700 mbar of He, resulting in an overall efficiency of 10%; *I*_t_: 150 mbar of Ar and 850 mbar of He, with 70% efficiency). The spectra were measured with a step size equivalent to 0.25 eV. Data were normalized using the program Athena^18^ with a linear pre-edge and polynomial postedge background subtracted from the raw ln(*I*_t_/*I*_0_) data. The samples were prepared in the form of a self-supported pellet with the thickness optimized to obtain an edge jump close to 1.

### Electrochemical Characterization

The electrode material was formed from a mixture of active material, electronically conductive carbon black C-NERGY Super C65 (Imerys Graphite & Carbon, Belgium), and polyvinylidene fluoride (MTI Corporation, USA) as a binder, in a ratio of 8:1:1. The materials were ground using an Agate pestle and mortar for 15 min. A slurry was made by adding NMP (*N*-methyl-2-pyrrolidone) (Merck, Germany) and mixed using a Thinky ARE-250 mixer (Intertronics, UK). The slurry was cast on carbon-coated aluminum foil using an MTI MSK-AFA-L800 tape caster (MTI Corporation, USA) and dried at 80 °C before being transferred to an 80 °C vacuum oven for a minimum of 16 h. Cathodes were cut to 12 mm using an MTI disc cutter (MTI Corporation, USA). CR2032 SS316 coin cells were assembled using the cathodes, 16 mm separators cut from the Whatman glass microfiber (GF/F grade) (Merck, Germany), and precut 15.6 mm lithium chips of 0.25 mm thickness (Cambridge Energy Solutions Ltd., UK) were used as the anode. The electrolyte was 1 M LiPF_6_ in ethylene carbonate and ethyl methyl carbonate, 3:7 v/v (Solvionic, France). Cyclic voltammetry (CV) measurements were conducted using a Biologic VMP-300 potentiostat at room temperature, and the galvanostatic cycling measurements were conducted using a MACCOR Series 4000 analyzer (Maccor, USA) at 25 °C.

## Results

### Structural Characterization of Li_3_Fe_3–*x*_In_*x*_TeO_12_ (*x* = 0, 1)

**Figure 1:**
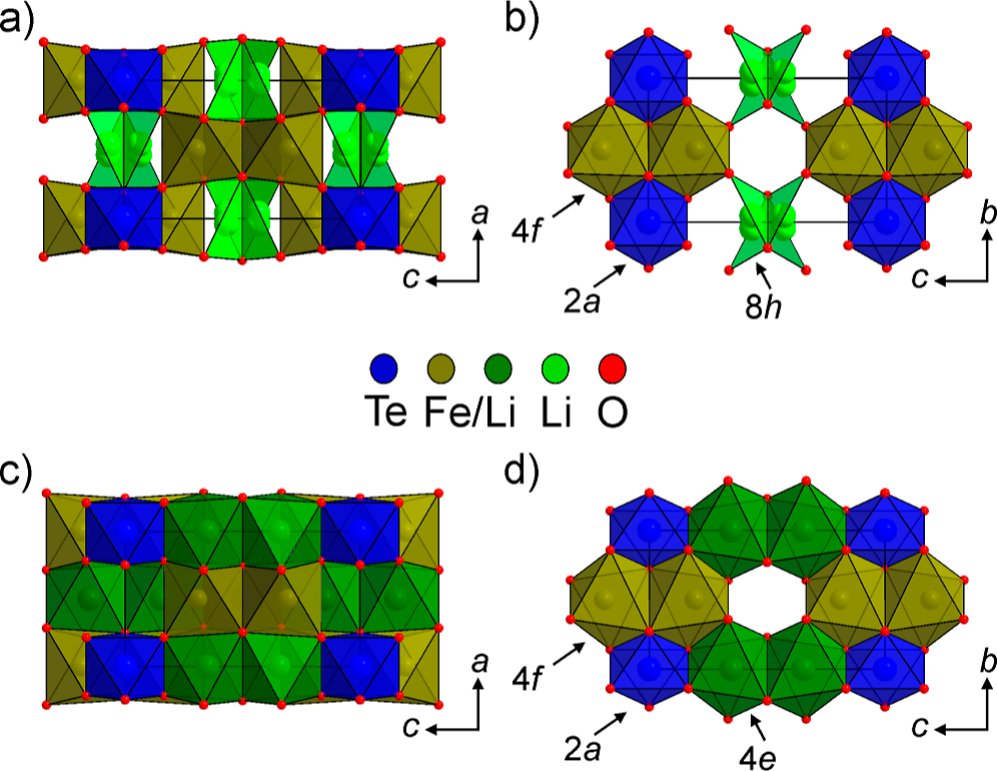
(a) Crystal structure of Li_3_Fe_3_Te_2_O_12_; (b) *bc*-plane of Li_3_Fe_3_Te_2_O_12_ indicating occupied cation sites within the hexagonal close-packed oxide framework; (c) crystal structure of Li_3+*x*_Fe_3_Te_2_O_12_; and (d) *bc*-plane of Li_3+*x*_Fe_3_Te_2_O_12_ indicating occupied cation sites within the hexagonal close-packed oxide framework.

**Figure 2:**
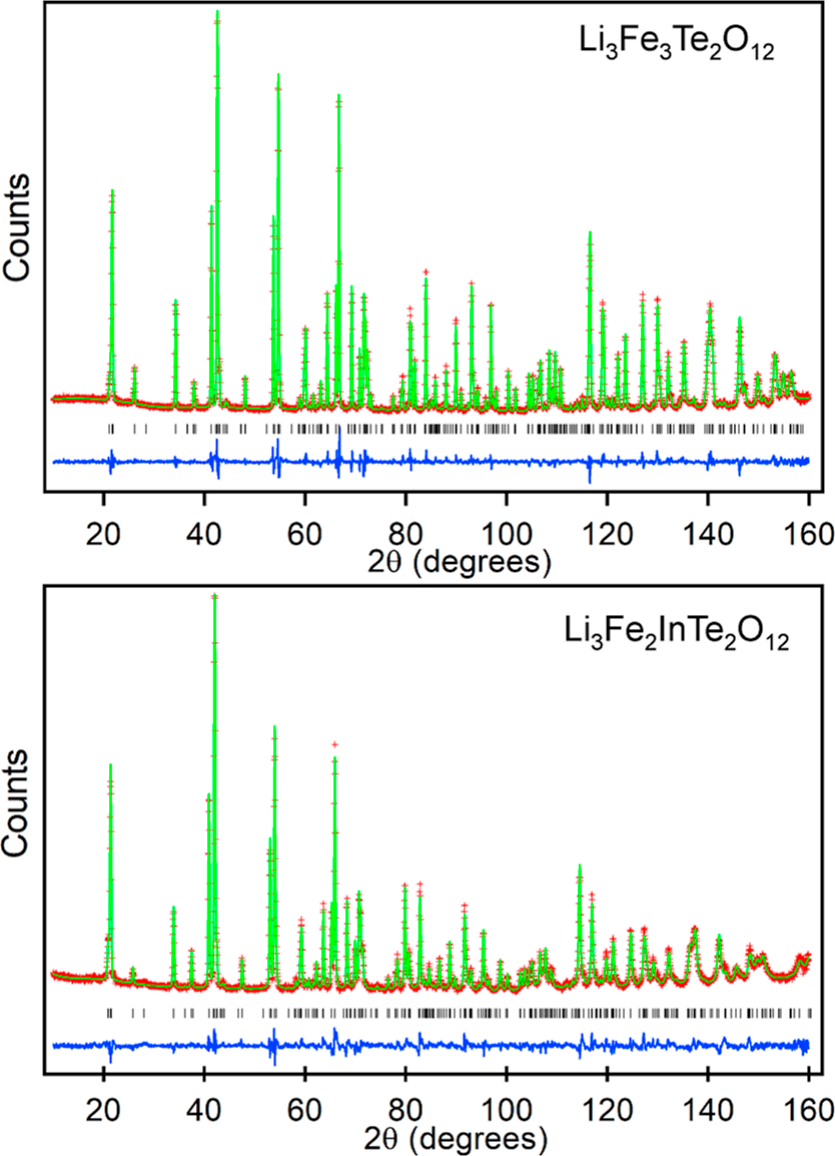
Observed calculated and difference plots from the structural refinement of Li_3_Fe_3_Te_2_O_12_ (top) and Li_3_Fe_2_InTe_2_O_12_ (bottom) vs NPD data collected at room temperature.

NPD data collected from Li_3_Fe_3_Te_2_O_12_ could be indexed using an orthorhombic unit cell (*a* = 4.911 Å, *b* = 5.084 Å, *c* = 8.427 Å) with reflection conditions consistent with *Pnnm* (no. 58) space group symmetry. Given the similarity of this phase to LiFe_2_SbO_6_^16^ and LiSbO_3_,^19^ a structural model was constructed, consisting of a hexagonally close-packed array of oxide ions with Te^6+^ and Fe^3+^ and Li^+^ cations residing in octahedral sites within the framework. The best fit to the data was achieved by locating the Te^6+^ cations on 2a octahedral sites and a 3:1 mixture of Fe^3+^ and Li^+^ cations on 4f octahedral sites, with the remaining Li^+^ cations distributed with 50% occupancy across an 8h site, which sits in the face of a tetrahedral site, as shown in Figure 1a,b. This model was refined against the NPD data to give a good fit, as shown in Figure 2, with a complete description of the refined structure and selected bond lengths detailed in the Supporting Information.

NPD data collected from Li_3_Fe_2_InTe_2_O_12_ could also be indexed by an orthorhombic cell with reflection conditions consistent with *Pnnm* (no. 58) space group symmetry. Thus, a model analogous to Li_3_Fe_3_Te_2_O_12_ (but with ^1^/_3_ of the Fe^3+^ cations on the 4f site replaced by In^3+^) was refined against the NPD data to achieve a good fit, as shown in Figure 2, with a complete description of the refined structure and selected bond lengths detailed in the Supporting Information.

### Characterization of Li_3_Fe_3–*x*_Al_*x*_Te_2_O_12_

PXRD data collected from samples in the composition range Li_3_Fe_3–*x*_Al_*x*_Te_2_O_12_ (0 < *x* < 0.6) revealed that single-phase samples could be prepared for samples with substitution levels up to *x* = 0.4, with larger substitution levels resulting in the formation of secondary phases, most likely Li_4.5_Al_0.5_TeO_6_.^20^ Thus, attention was focused on the *x* = 0.4 composition.

SXRD data collected from Li_3_Fe_2.6_Al_0.4_Te_2_O_12_ could be indexed by an orthorhombic unit cell (*a* = 4.922 Å, *b* = 5.096 Å, *c* = 8.451 Å) with reflection conditions consistent with the *Pnnm* (no. 58) space group. Thus, a model based on the structure of Li_3_Fe_3_Te_2_O_12_ was constructed with 13.3% of the Fe replaced by Al and refined against the SXRD data. The refinement converged readily to achieve a good fit, with full structural details described in the Supporting Information.

### Chemical Lithiation of Li_3_Fe_3–*x*_M_*x*_Te_2_O_12_ Samples

To investigate the lithium insertion behavior of the Li_3_Fe_3–*x*_M_*x*_Te_2_O_12_ (M = In, Al) phases, samples were reacted with *n*-BuLi as described above. We took this approach using chemical lithiation rather than recovering material from electrochemical cells to simplify the structural analysis and avoid complications associated with the presence of other cell components (carbon, electrolyte, etc.) in samples.

The reactions between *n*-BuLi and Li_3_Fe_3_Te_2_O_12_ or Li_3_Fe_2.6_Al_0.4_Te_2_O_12_ had to be performed with great care to avoid the formation of elemental tellurium, as described in detail in the Supporting Information. It was observed that the reaction durations must be kept to less than 24 h to avoid the formation of elemental Te. This was not the case for reactions between *n*-BuLi and Li_3_Fe_2_InTe_2_O_12_, which appear to be more robust with respect to the reduction of tellurium.

SXRD data collected from Li_3+δ_Fe_3_Te_2_O_12_, Li_3+δ_Fe_2_InTe_2_O_12_, and Li_3+δ_Fe_2.6_Al_0.4_Te_2_O_12_ could all be indexed using orthorhombic cells with *Pnnm* space group symmetry but with cell volumes expanded by 5–7% compared to the corresponding unlithiated materials. Attempts to fit the SXRD data with structural models based on the “pristine” materials yielded poor fits to the data until some of the Fe cations were moved from the 4f site to an octahedral 4e (0, 0, *z* ∼ 0.65) site (with which the 4f site shares a face) close to the 8h site occupied by lithium in the pristine Li_3_Fe_3–*x*_In_*x*_Te_2_O_12_ phases, as shown in Figure 1c,d. Refinement of these models against the SXRD data revealed 26% of the Fe cations [0.77 Fe per formula unit (fu)] migrated to the 4e site on the formation of Li_3+δ_Fe_3_Te_2_O_12_, compared to 19% of the Fe cations (0.38 Fe per fu) for Li_3+δ_Fe_2_InTe_2_O_12_ and 6.4% (0.16 Fe per fu) for Li_3+δ_Fe_2.6_Al_0.4_Te_2_O_12._ Full details of these structural refinements are given in the Supporting Information. It was assumed that neither the In nor Al cations changed their coordination site on lithium insertion.

To investigate the chemical reversibility of the lithium insertion process, samples were reacted with iodine, as described above. SXRD data collected from an iodine-reacted sample of Li_3+δ_Fe_3_Te_2_O_12_ could be fit by the structural model used for the “pristine” Li_3_Fe_3_Te_2_O_12_ phase, with lattice parameters close to those of the unlithiated phases. In addition, the SXRD data showed no evidence for Fe-cations on the 4e coordination site (occupancy refined to zero), indicating that Fe-migration associated with lithium insertion is fully reversible.

In contrast, SXRD data collected from an iodine-reacted sample of Li_3+δ_Fe_2_InTe_2_O_12_ were better fit by a model that retained some Fe cations on the 4e site of the material (*R*_Bragg_ = 1.11 with Fe migration, *R*_Bragg_ = 1.50 without Fe migration). The Fe occupancy of the 4e site refined to a value of 0.088(1) for I_2_-treated Li_3+δ_Fe_2_InTe_2_O_12_, compared to a value of 0.095(1) prior to reaction with I_2_, indicating that the Fe-migration was largely irreversible in the indium-substituted phase.

The behavior of Li_3+δ_Fe_2.6_Al_0.4_Te_2_O_12_ on treatment with iodine appears intermediate between that of Li_3+δ_Fe_3_Te_2_O_12_ and Li_3+δ_Fe_2_InTe_2_O_12_. Refinement of a model including Fe cations on the 4e site fit the data better than one without (*R*_Bragg_ = 2.00 with Fe migration, *R*_Bragg_ = 2.44 without Fe migration) to yield a 4e site Fe occupancy of 0.018(1) for I_2_-treated Li_3+δ_Fe_2.6_Al_0.4_Te_2_O_12_, compared to a value of 0.041(1) prior to reaction with I_2_, indicating partial reversibility of the Fe-migration in this phase. A complete description of the refined structures of all of the iodine-treated samples is given in the Supporting Information.

**Figure 3:**
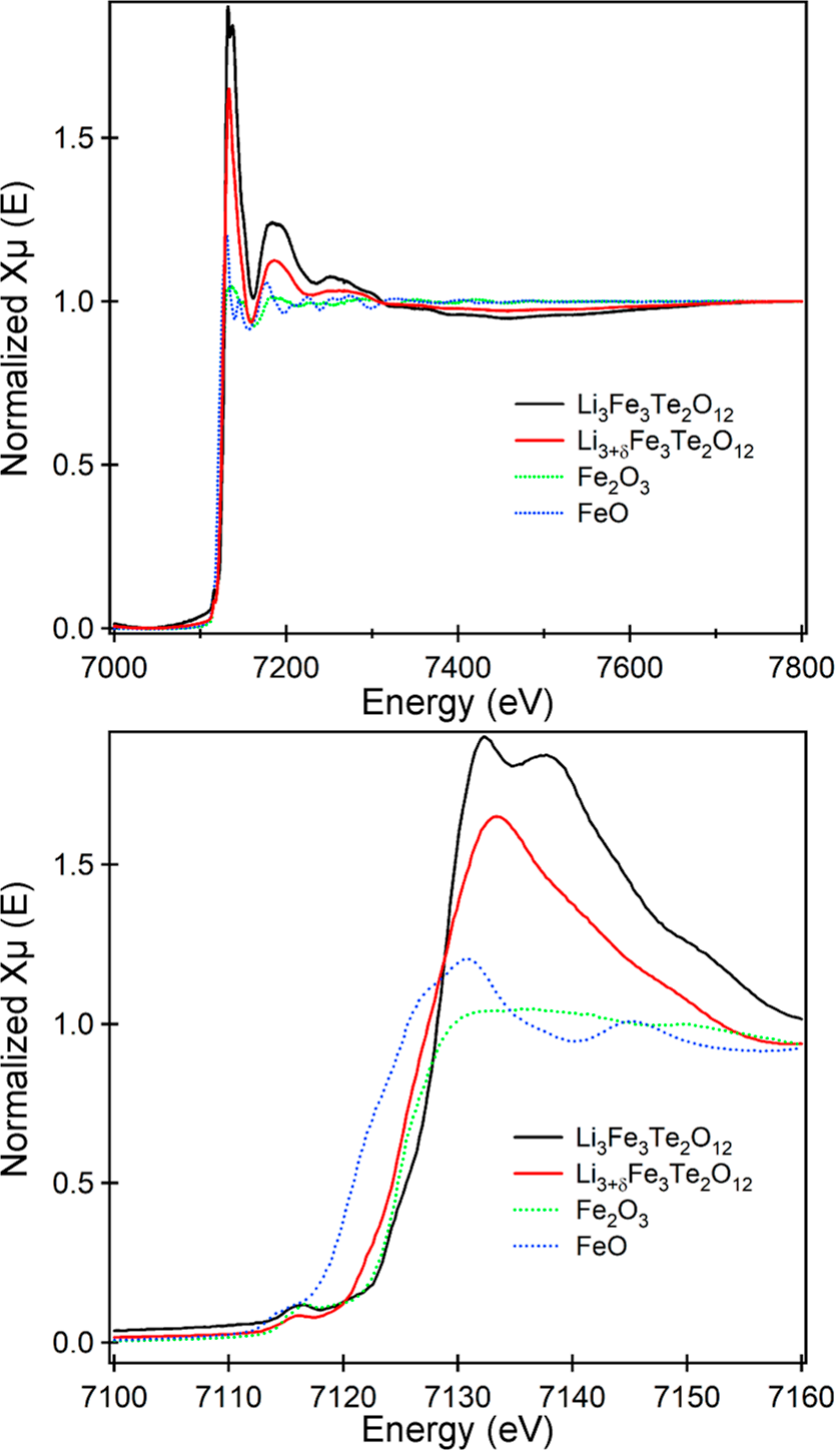
XAS data collected from “pristine” and lithiated Li_3_Fe_3_Te_2_O_12_ compared to data collected from Fe_2_O_3_ and FeO standards.

X-ray absorption spectra collected at the Fe K-edge from “pristine” Li_3_Fe_3_Te_2_O_12_ and lithiated Li_3+δ_Fe_3_Te_2_O_12_ are shown in Figure 3, in comparison to Fe^2+^ and Fe^3+^ standards. It can clearly be seen that on lithiation, the Fe K-edge moves to lower energy, consistent with the reduction of Fe^3+^ to Fe^2+^.

### Electrochemical Characterization

**Figure 4:**
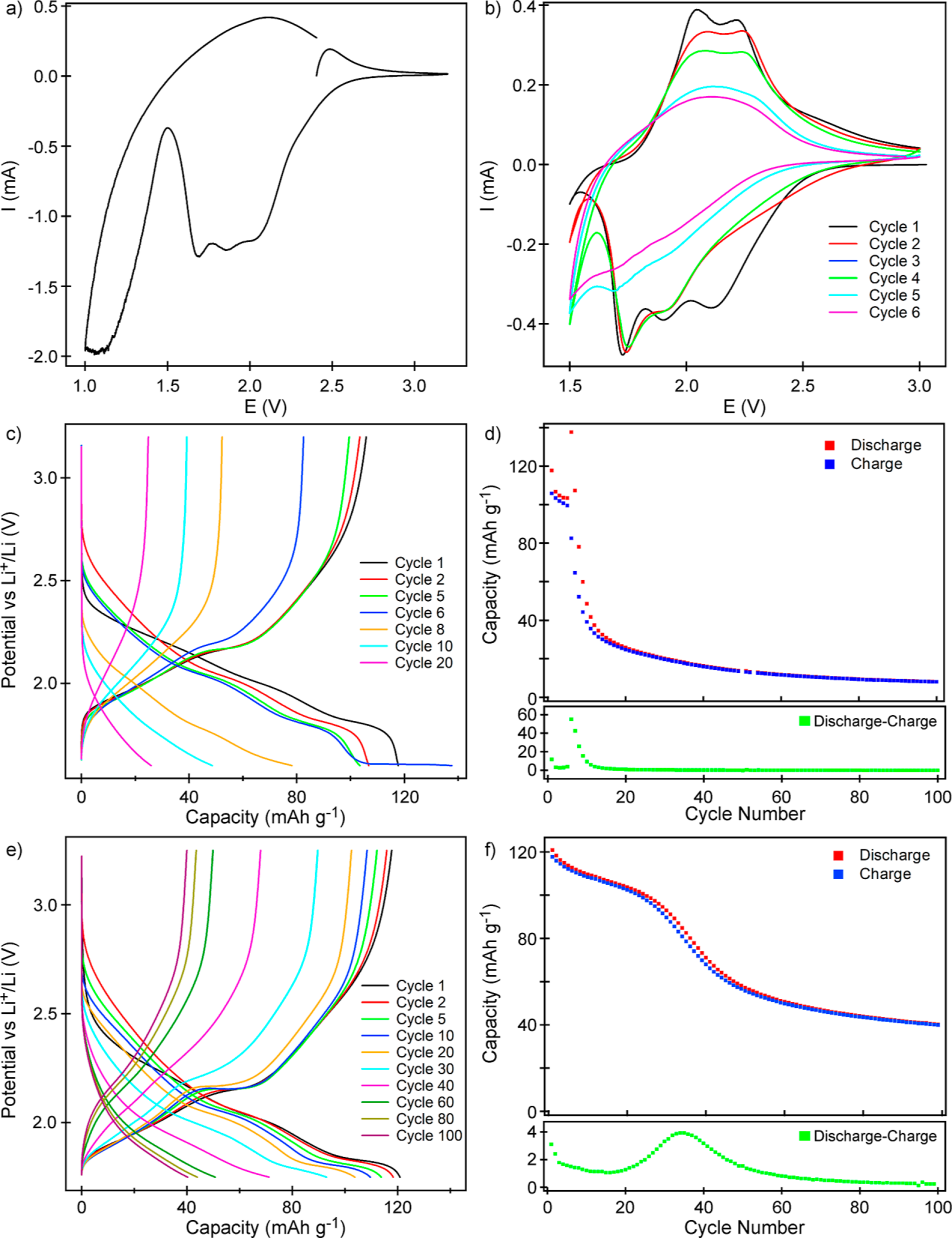
CV data were collected from Li_3_Fe_3_Te_2_O_12_ in the ranges (a) 1.0–3.25 and (b) 1.5–3 V. Discharge–charge cycling profiles (c) and capacity data (d) were collected from Li_3_Fe_3_Te_2_O_7_ in the range of 3.15–1.6 V from half coin cells. Discharge–charge cycling profiles (e) and capacity data (f) were collected from Li_3_Fe_3_Te_2_O_7_ in the range of 3.25–1.75 V.

CV data were collected from pristine samples of Li_3_Fe_3_Te_2_O_12_ in the range 1–3.25 V, as shown in Figure 4a. On sweeping the potential down from 3.25 V, two broad reduction features are observed. The first, between 2.5 and 1.5 V, is assigned to the reduction of Fe^3+^ to Fe^2+^ on lithium insertion. The second feature, between 1.5 and 1 V, is assigned to the reduction of Te^6+^ to elemental Te, accompanied by the decomposition of the bulk Li_3_Fe_3_Te_2_O_12_ phase. This latter assignment is consistent with the observation noted above that the reaction of *n*-BuLi (*E* = +1.0 V vs Li/Li^+^)^21^ with Li_3_Fe_3_Te_2_O_12_ results in the formation of elemental Te over extended reaction times.

To better understand the electrochemical cycling behavior of Li_3_Fe_3_Te_2_O_12_, CV data were collected from a pristine sample in the range 1.5–3 V, as shown in Figure 4b. These data show that on sweeping the potential down from 3 V, a sequence of three distinct reductive maxima is observed at 2.1, 1.9, and 1.7 V. On the reverse voltage sweep from 1.5 to 3 V, two distinct oxidation events are observed at 2.05 and 2.2 V. We attribute the multiple redox features observed on the first cycle to the reversible site migration of Fe and Li cations on Li insertion/extraction, as observed for the chemically reduced and reoxidized samples described above. On the second voltage cycle, the reductive feature at 2.1 V becomes less well resolved, and a new, strong reductive event is observed below 1.6 V. This latter feature strengthens on subsequent cycles and is accompanied by an overall decline in the magnitude and distinctiveness of both reductive and oxidative processes. We again attribute the reductive feature below 1.6 V to the reduction of Te^6+^ cations in the cathode framework, leading to the extrusion of elemental tellurium and the decomposition of the cathode material, accounting for the dramatic loss of electrochemical activity on subsequent voltage cycles.

Capacity data collected during discharge–charge cycling of Li_3_Fe_3_Te_2_O_12_ in the range 3.15–1.6 V (Figure 4c,d) reveal an initial discharge capacity of 118 mA h g^–1^ (2.80 Li per fu) principally in the voltage range 2.4–1.75 V. The subsequent charging step yielded a capacity of 105 mA h g^–1^ (2.49 Li per fu), with Li extraction occurring at potentials greater than 1.85 V. Thus, the first discharge–charge cycle agrees well with the CV data shown in Figure 4b.

Subsequent discharge–charge cycles show a steady decline in capacity over 5 cycles to a value of ∼100 mA h g^–1^. However, the sixth discharge shows a dramatic rise in the insertion capacity to 137 mA h g^–1^ (3.25 Li per fu). Close inspection of the potential-capacity trace reveals the initial discharge profile of the sixth cycle, which looks similar to previous cycles, discharging ∼100 mA h g^–1^ (2.35 Li per fu) down to 1.65 V. However, at potentials between 1.65 and 1.6 V, a further 37 mA h g^–1^ (0.9 Li per fu) appears to be “inserted”. We interpret this additional 0.9 Li per fu as being due to the reduction of Te^6+^ to elemental Te and the associated sample decomposition. This interpretation is consistent with a charging capacity of only 82 mA h g^–1^ (1.95 Li per fu) recorded for the subsequent charging step and a rapid decline in cycling capacity over the following four cycles to around ∼40 mA h g^–1^ (∼1 Li per fu). The decomposition of the cathode material via Te^6+^ reduction can be seen clearly via the “excess inserted Li” (i.e., the difference between the discharge and charge capacity for each cycling step) for cycles 6–10 shown in the bottom panel in Figure 4d.

The reduction of Te^6+^, which leads to cathode decomposition, occurs at low potential (*E* < 1.65 V), so electrochemical discharge–charge cycling was performed in the range of 3.25–1.75 V in an attempt to limit this. Figure 4e,f shows an initial discharge capacity of 120 mA h g^–1^ (2.85 Li per fu) and a subsequent charge of 117 mA h g^–1^ in this voltage range. The discharge capacity slowly declines to 103 mA h g^–1^ (2.44 Li per fu) over 20 cycles before dropping more rapidly over the next 30 cycles to ∼55 mA h g^–1^ (1.3 Li per fu), with an associated increase in “excess inserted Li” in these cycles. This suggests that the cathode material is again decomposing via the reduction of Te^6+^, further suggesting that this decomposition pathway cannot be fully suppressed by changing the voltage window in which the cathode operates.

**Figure 5:**
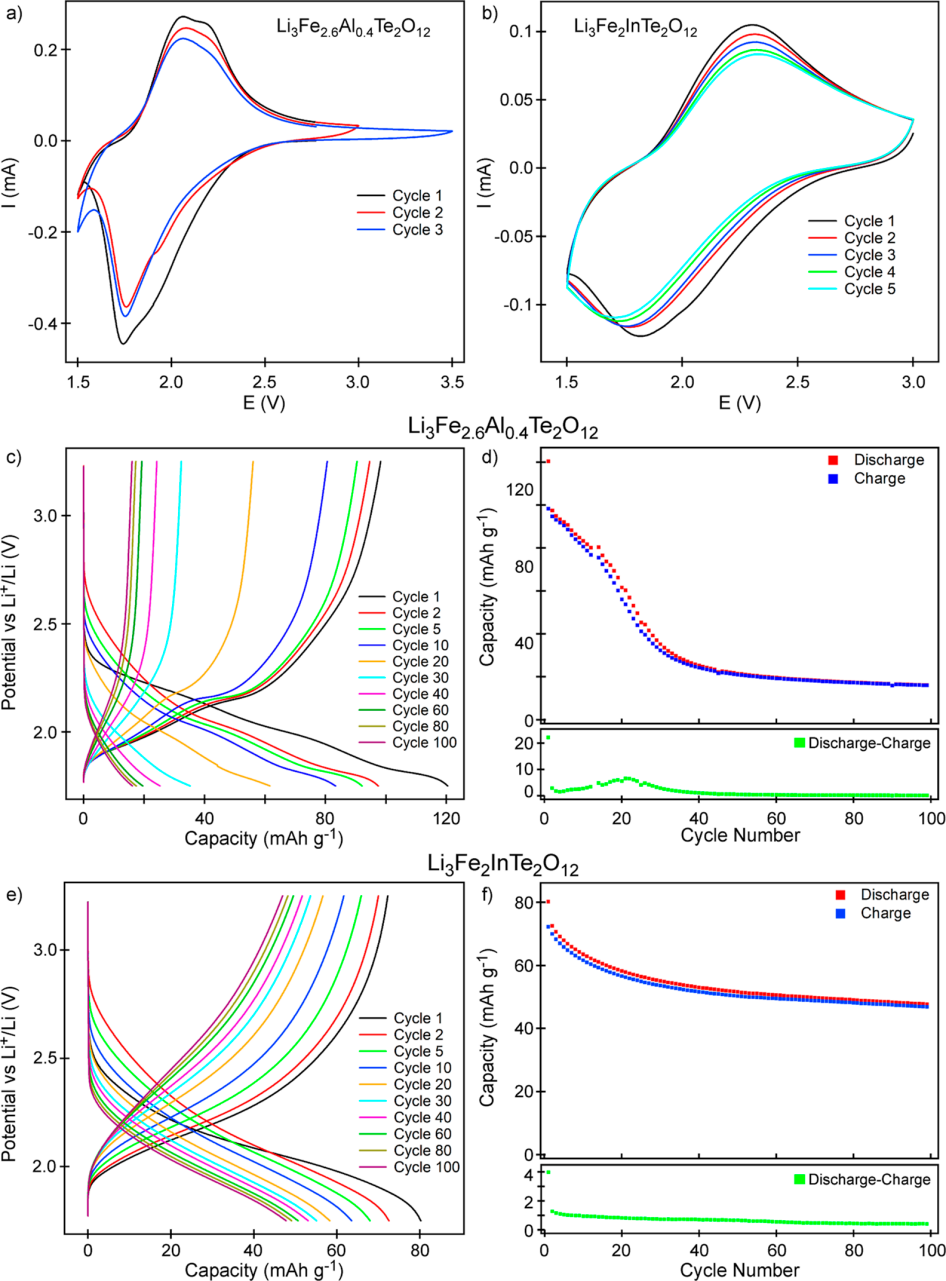
CV data were collected from (a) Li_3_Fe_2.6_Al_0.4_Te_2_O_12_ in the range of 1.5–3.5 V and (b) Li_3_Fe_2_InTe_2_O_12_ in the range of 1.5–3.0 V from half coin cells. Discharge–charge cycling profiles (c) and capacity data (d) were collected from Li_3_Fe_2.6_Al_0.4_Te_2_O_12_ in the range of 3.25–1.75 V. Discharge–charge cycling profiles (c) and capacity data (d) were collected from Li_3_Fe_2_InTe_2_O_12_ in the range of 3.25–1.75 V.

CV data collected from Li_3_Fe_2.6_Al_0.4_Te_2_O_12_ (Figure 5a) are qualitatively similar to analogous data from Li_3_Fe_3_Te_2_O_12_, exhibiting a broad reduction feature maximum at 1.75 V and a broad oxidation feature spanning 1.8–2.4 V. In addition, the second and third cycles show a further reduction feature developing below 1.6 V, suggesting that the reduction of Te^6+^ is occurring. Discharge–charge capacity data in the range 1.75–3.25 V (Figure 5c,d) appear to confirm this. The initial discharge appears to be slightly anomalous, exhibiting a capacity of 120 mA h g^–1^ (2.79 Li per fu), which is greater than the maximum capacity of the Fe^3+/2+^ reduction (111 mA h g^–1^, 2.6 Li per fu), suggesting that some noninsertion reduction processes are occurring. The second cycle exhibits a discharge capacity of 97 mA h g^–1^ (2.26 Li per fu), which drops to 80 mA h g^–1^ over 13 cycles and then rapidly to ∼30 mA h g^–1^ over the next 20 cycles, with a maximum in the excess lithium insertion at cycle 21, consistent with cathode decomposition via Te^6+^ reduction. Thus, we conclude that Al-for-Fe cation substitution does not stabilize the Li_3_Fe_3_Te_2_O_12_ system to Te-reduction-decomposition.

CV data collected from Li_3_Fe_2_InTe_2_O_12_ between 1.5 and 3 V are qualitatively different from analogous data from Li_3_Fe_3_Te_2_O_12_. They exhibit a broad reduction feature centered at 1.8 V and a broad oxidation feature centered at 2.3 V. There is no sign of a reduction feature attributable to Te^6+^, even after multiple voltage sweeps. Capacity data collected during discharge–charge cycles reveal a first discharge capacity of 80 mA h g^–1^ (2.07 Li per fu), which steadily declines so that after 20 cycles, it is 58 mA h g^–1^ (1.5 Li per fu) and 47 mA h g^–1^ (1.22 Li per fu) after 100 cycles in half coin cells. No significant maximum is observed in the excess inserted lithium plot after the first cycle. These data show no indication of the Te^6+^ reduction observed for Li_3_Fe_3_Te_2_O_12_ and Li_3_Fe_2.6_Al_0.4_Te_2_O_12_ at low potential, demonstrating that In-for-Fe substitution suppresses this decomposition pathway.

## Discussion

### Influence of Substitution on Cation Migration

Electrochemical lithium insertion into Li_3_Fe_3_Te_2_O_12_, using a potential of 1.75 V vs Li/Li^+^, intercalates ∼2.85 lithium atoms per fu in the first cycle, reducing the Fe^3+^ centers to an average oxidation state of Fe^+2.05^. The reduction potential of *n*-BuLi in MeCN is ∼+1.0 V vs Li/Li^+^,^21^ suggesting that the chemically reduced Li_3+δ_Fe_3_Te_2_O_12_ samples have compositions close to Li_6_Fe_3_Te_2_O_12_. Crystallographic analysis of chemically lithiated Li_3+δ_Fe_3_Te_2_O_12_ reveals that Li insertion is accompanied by the migration of 26% of the Fe cations (0.77 Fe per fu) from the 4f site to a 4e site, as shown in Figure 1, with the Li ions filling the remaining vacancies on the 4f and 4e sites, to give an overall composition of Li_6_Fe_3_Te_2_O_12_ if all the sites are filled.

Examining the crystal structure of pristine Li_3_Fe_3_Te_2_O_12_ reveals that a Fe cation on the 4f site of the material has a bond valence sum (BVS)^22,23^ of Fe + 2.69 (Table S4) and thus has Fe–O bond lengths that are a little longer than optimum for Fe^3+^ cations. On reduction, the average M–O bond length of the 4f site increases from 2.060 Å in Li_3_Fe_3_Te_2_O_12_ to 2.165 Å in Li_3+δ_Fe_3_Te_2_O_12_, corresponding to a BVS of Fe + 1.99 in the reduced phase. Furthermore, the 4e sites onto which the Fe cations migrate in Li_3+δ_Fe_3_Te_2_O_12_ have an average M–O bond length of 2.194 Å, corresponding to a BVS of Fe + 1.94. Thus, it can be seen that on lithium insertion, the Li_3+δ_Fe_3_Te_2_O_12_ framework adjusts to accommodate the Fe centers in coordination environments suitable for Fe^2+^ without changing the TeO_6_ coordination polyhedra significantly (Li_3_Fe_3_Te_2_O_12_ ⟨Te–O⟩ = 1.919 Å; Li_3+δ_Fe_3_Te_2_O_12_, ⟨Te–O⟩ = 1.924 Å). It appears likely that the migration of the Fe cations plays a key part in facilitating this structural change on reduction, as has been observed in other Li–Fe–M–O phases, such as Li_1+δ_Fe_2_SbO_6_.^16^ Chemical delithiation using I_2_ appears to completely reverse both the lithium insertion (as assessed by the lattice parameters of the I_2_-treated phase) and the Fe migration. This complete reversibility contrasts strongly with the behavior observed for Li_1+*x*_Fe_2_SbO_6_, where the presence of a cation-ordered and Fe^2+^/Fe^3+^ charge-ordered state prevents lithium extraction.^16^ We attribute the differing behavior of LiFe_2_SbO_6_ and Li_3_Fe_3_Te_2_O_12_ to the subtle change in structure and Li–Fe–M ordering that occurs on switching from Sb^5+^ to Te^6+^, which allows 1 Li to be inserted per Fe center in Li_3_Fe_3_Te_2_O_12_ (rather than 0.5 Li per Fe in LiFe_2_SbO_6_) and thus avoids a Fe^2+/3+^ charge-ordered state analogous to that seen in Li_2_Fe_2_SbO_6_.

In-for-Fe substitution to form Li_3_Fe_2_InTe_2_O_12_ leads to a unit cell expansion of 4.6%. The cell expansion appears to be driven by a significant increase in the size of the 4f Fe/In/Li coordination site (⟨M–O⟩ = 2.099 Å, BVS = Fe + 2.44) consistent with the larger ionic radius of In^3+^ (0.800 Å) compared to that of Fe^3+^ (0.645 Å).^13^ The electrochemical reduction of Li_3_Fe_2_InTe_2_O_12_ indicates ∼2 Li atoms per fu are intercalated at a potential of 1.75 V. This suggests that the chemically reduced Li_3+δ_Fe_2_InTe_2_O_12_ phases have compositions close to Li_5_Fe_2_InTe_2_O_12_ and average iron oxidation states of Fe^2+^. Upon lithium insertion, the 4f coordination site further expands (⟨M–O⟩ = 2.174 Å; BVS = Fe + 2.02), and a small fraction of the Fe cations (19% of Fe, 0.38 Fe per fu) migrate to the 4e site, which is of a suitable size to accommodate Fe^2+^ (⟨M–O⟩ = 2.211 Å, BVS = Fe + 1.99). The smaller degree of cation migration in the In-substituted material can be rationalized on the basis of the larger 4f site in unlithiated Li_3_Fe_2_InTe_2_O_12_ needing to expand less on Li insertion and also the lower absolute level of Li insertion in Li_3+δ_Fe_2_InTe_2_O_12_ (∼2 Li per fu) compared to Li_3+δ_Fe_3_Te_2_O_12_ (∼3 Li per fu). Reaction with I_2_ appears to remove the majority of the inserted lithium (based on lattice parameter changes); however, the cation migration appears to be irreversible in the In-substituted phase.

Al-for-Fe substitution to form Li_3_Fe_2.6_Al_0.4_Te_2_O_12_ results in a small (0.8%) cell expansion despite the smaller ionic radius of Al^3+^ (0.535 Å) compared to Fe^3+^ (0.645 Å).^13^ The low level of Al-substitution achieved results in only small changes to the average crystal structure of the Li_3_Fe_3–*x*_Al_*x*_Te_2_O_12_ material, with the size of the 4f coordination site remaining effectively unchanged (⟨M–O⟩ = 2.058 Å, BVS = Fe + 2.72) compared to Li_3_Fe_3_Te_2_O_12_. It is, therefore, hard to explain why this level of Al-for-Fe substitution suppresses Fe migration on Li insertion on the basis of average coordination site sizes, suggesting that local distortions may play an important role.

### Influence of Substitution on Redox Potentials and Stability

Unsubstituted Li_3_Fe_3_Te_2_O_12_ can be electrochemically cycled, making use of the Fe^3+^/Fe^2+^ redox couple, giving an initial capacity of 120 mA h g^–1^, corresponding to the insertion of ∼2.85 Li per fu, close to the maximum theoretical capacity (limited by the Fe^3+^/Fe^2+^ redox couple and availability of Li coordination sites) of 126 mA h g^–1^ (3 Li per fu). However, the cathode material exhibits poor long-term cycling capacity due to the redox instability of Te^6+^, which is reduced to elemental Te at potentials below ∼1.6 V vs Li/Li^+^. This instability leads to a dramatic loss of capacity after 6 cycles when operating in the potential range 1.6–3.15 V and ∼20 cycles when cycling between 1.75 and 3.25 V. This operating-potential-dependent delay to the decomposition process suggests that electrochemical cycling activates the Te^6+^/Te reduction (presumably by modifying the particle size or morphology), raising the potential at which it occurs into the selected operating voltage range. Thus, we conclude that the proximity of the Te^6+^/Te (<1.6 V) and Fe^3+^/Fe^2+^ (1.65–2.2 V) redox couples in Li_3_Fe_3_Te_2_O_12_ means that even careful selection of operating potentials cannot easily prevent cathode decomposition via Te^6+^ reduction over multiple cycles.

As noted above, the level of Al-for-Fe substitution, which can be achieved in Li_3_Fe_3–*x*_Al_*x*_Te_2_O_12_ phases, is quite small. As a consequence, the effect of this substitution on the redox potentials and cycling stability of Al-substituted cathode materials is small, resulting in behavior that is qualitatively similar to that of the unsubstituted materials. However, In-for-Fe substitution raises the Fe^3+^/Fe^2+^ redox couple to ∼1.8 V in Li_3_Fe_2_InTe_2_O_12_. This change can be attributed to the expansion of the 4f coordination site (described above), stabilizing the reduced form of Li_3+δ_Fe_2_InTe_2_O_12_ compared to that of Li_3+δ_Fe_3_Te_2_O_12_. In addition, In substitution appears to prevent Te^6+^ reduction, even after 100 cycles. It is not clear what the origin of this stabilization is, as the 2*a* TeO_6_ coordination sites in Li_3_Fe_3_Te_2_O_12_, Li_3_Fe_2_InTeO_12_, and their lithiated products are remarkably similar. However, as a consequence of this stabilization, Te^6+^ reduction is avoided when cycling to 1.6 V.

## Conclusions

Li_3_Fe_3_Te_2_O_12_ adopts a LiSbO_3_-like structure in which the metal cations reside in octahedral coordination sites within a hexagonally close-packed array of oxide ions. Approximately 3 lithium atoms per formula unit can be reversibly inserted, either chemically or electrochemically, into Li_3_Fe_3_Te_2_O_12_ in a process accompanied by significant Fe-cation migration. This behavior contrasts strongly with that of LiFe_2_SbO_6_, another Li–Fe–M–O phase with a LiSbO_3_-related structure, into which only 1 lithium per formula unit can be inserted in an irreversible process, as Li_2_Fe_2_SbO_6_ adopts a cation-ordered and Fe^2+/3+^ charge-ordered state, which prevents lithium extraction. By switching from Sb to Te, a different ratio and distribution of cations are adopted within the hcp framework of oxide ions, facilitating the electrochemical cycling of the Te-containing material. However, a side effect of switching from Sb to more electronegative Te is that the system becomes unstable with respect to decomposition via the reduction of Te^6+^, limiting long-term cycling capacity. Partial substitution of Fe^3+^ by In^3+^ reduces the degree of Fe-cation migration and suppresses the reduction of Te^6+^, such that Li_3_Fe_2_InTe_2_O_12_ shows no evidence of this decomposition pathway even after 100 cycles. Unfortunately, replacing electrochemically active Fe^3+^ with inactive In^3+^ lowers the capacity of the material, so that while Li_3_Fe_2_InTe_2_O_12_ exhibits a much smaller fractional capacity loss on cycling, the absolute capacity of Li_3_Fe_2_InTe_2_O_12_ only exceeds that of the all-iron compound after 60 cycles, highlighting the ongoing challenges to developing competitive Fe-based Li-ion cathode materials.

## Supporting Information Available

Complete description of the structural refinements of Li_3_Fe_3_Te_2_O_12_, Li_3_Fe_2_InTe_2_O_12_, Li_3_Fe_2.6_Al_0.4_Te_2_O_12_, and their lithium-inserted and I_2_-treated products (PDF)
