# Supplementary material for: Influence of Cation Substitution on Cycling Stability and Fe-Cation Migration in Li3Fe3–xMxTe2O12 (M = Al, In) Cathode Materials
Source: Inorg Chem. 2024 Jan 4;63(2):1395–403. doi: 10.1021/acs.inorgchem.3c03929 (PMC10792598; doi:10.1021/acs.inorgchem.3c03929)
Supplement: Supplementary file 1 — ic3c03929_si_001.pdf [file ic3c03929_si_001.pdf]

Influence of cation substitution on cycling stability and Fe-cation migration in $Li_3Fe_{3-x}M_xTe_2O_{12}$ (M = Al, In) cathode materials.

Xabier Martínez de Irujo Labalde [†Ψ], Man Yi Lee [†Ψ], Heather Grievson [ΨΧ], Josie-May Mortimer [ΨΧ], Samuel G. Booth [ΨΧ], Emmanuelle Suard [§], Serena A. Cussen [ΨΧ] and Michael A. Hayward [†Ψ*].

[†] Department of Chemistry, University of Oxford, Inorganic Chemistry Laboratory, South Parks Road, Oxford, OX1 3QR, UK.

[Ψ] The Faraday Institution, Quad One, Harwell Campus, Didcot, OX11 0RA, UK.

[Χ] Department of Materials Science and Engineering, Sir Robert Hadfield Building, University of Sheffield, Sheffield S1 3JD, UK

[§] Institut Laue-Langevin - 71 avenue des Martyrs 38000 Grenoble - France.

Supporting Information

Table of Contents

1. Structural characterisation of $Li_3Fe_3Te_2O_{12}$, $Li_{3+\delta}Fe_3Te_2O_{12}$ and iodine-oxidized $Li_{3+\delta}Fe_3Te_2O_{12}$.

Figure S1. Observed, calculated and difference plots from the structural refinement of $Li_3Fe_3Te_2O_{12}$ against NPD data collected at room temperature.

Table S1. Parameters from the structural refinement of $Li_3Fe_3Te_2O_{12}$ against NPD data collected at room temperature.

Figure S2. Observed, calculated and difference plots from the structural refinement of $Li_{3+\delta}Fe_3Te_2O_{12}$ against SXRD data collected at room temperature.

Table S2. Parameters from the structural refinement of $Li_{3+\delta}Fe_3Te_2O_{12}$ against SXRD data collected at room temperature.

Figure S3. Observed, calculated and difference plots from the structural refinement of $Li_{3+\delta}Fe_3Te_2O_{12}$ which had been reacted with I_2, against SXRD data collected at room temperature.

Table S3. Parameters from the structural refinement of $Li_{3+\delta}Fe_3Te_2O_{12}$, which had been treated with I_2, against SXRD data collected at room temperature.

Table S4. Selected bond lengths (Å) from the refined structures of $Li_3Fe_3Te_2O_{12}$, $Li_{3+\delta}Fe_3Te_2O_{12}$ and iodine-treated $Li_{3+\delta}Fe_3Te_2O_{12}$.

2. Structural characterisation of $Li_3Fe_2InTe_2O_{12}$, $Li_{3+\delta}Fe_2InTe_2O_{12}$ and iodine-oxidized $Li_{3+\delta}Fe_2InTe_2O_{12}$.

Figure S4. Observed, calculated and difference plots from the structural refinement of $Li_3Fe_3Te_2O_{12}$ against NPD data collected at room temperature.

Table S5. Parameters from the structural refinement of $Li_3Fe_2InTe_2O_{12}$ against NPD data collected at room temperature.

Figure S5. Observed, calculated and difference plots from the structural refinement of $Li_{3+\delta}Fe_2InTe_2O_{12}$ against SXRD data collected at room temperature.

Table S6. Parameters from the structural refinement of $Li_{3+\delta}Fe_2InTe_2O_{12}$ against SXRD data collected at room temperature.

Figure S6. Observed, calculated and difference plots from the structural refinement of $Li_{3+\delta}Fe_2InTe_2O_{12}$ which had been reacted with I_2, against SXRD data collected at room temperature.

Table S7. Parameters from the structural refinement of $Li_{3+\delta}Fe_2InTe_2O_{12}$, which had been treated with I_2, against SXRD data collected at room temperature.

Table S8. Selected bond lengths (Å) from the refined structures of $Li_3Fe_2InTe_2O_{12}$, $Li_{3+\delta}Fe_2InTe_2O_{12}$ and iodine-treated $Li_{3+\delta}Fe_2InTe_2O_{12}$.

3. Structural characterisation of $Li_3Fe_{2.6}Al_{0.4}Te_2O_{12}$, $Li_{3+\delta}Fe_{2.6}Al_{0.4}Te_2O_{12}$ and iodine-oxidized $Li_{3+\delta}Fe_{2.6}Al_{0.4}Te_2O_{12}$.

Figure S7. Observed, calculated and difference plots from the structural refinement of $Li_3Fe_{2.6}Al_{0.4}Te_2O_{12}$ against SXRD data collected at room temperature.

Table S9. Parameters from the structural refinement of $Li_3Fe_{2.6}Al_{0.4}Te_2O_{12}$ against SXRD data collected at room temperature.

Figure S8. Observed, calculated and difference plots from the structural refinement of $Li_{3+\delta}Fe_{2.6}Al_{0.4}Te_2O_{12}$ against SXRD data collected at room temperature.
Table S10. Parameters from the structural refinement of $Li_{3+\delta}Fe_{2.6}Al_{0.4}Te_2O_{12}$ against SXRD data collected at room temperature.
Figure S9. Observed, calculated and difference plots from the structural refinement of $Li_{3+\delta}Fe_{2.6}Al_{0.4}Te_2O_{12}$ which had been reacted with I_2, against SXRD data collected at room temperature.
Table S11. Parameters from the structural refinement of $Li_{3+\delta}Fe_{2.6}Al_{0.4}Te_2O_{12}$, which had been treated with I_2, against SXRD data collected at room temperature.
Table S12. Selected bond lengths (Å) from the refined structures of $Li_3Fe_2InTe_2O_{12}$, $Li_{3+\delta}Fe_2InTe_2O_{12}$ and iodine-treated $Li_{3+\delta}Fe_2InTe_2O_{12}$.

4. Chemical lithiation of $Li_3Fe_3Te_2O_{12}$ and $Li_3Fe_{2.6}Al_{0.4}Te_2O_{12}$.

Figure S10. PXRD data collected from a sample of $Li_3Fe_3Te_2O_{12}$ exposed to *n*-BuLi for 10 days. Arrows indicate diffraction peaks due to elemental Te.

1. Structural characterisation of $Li_3Fe_3Te_2O_{12}$, $Li_{3+\delta}Fe_3Te_2O_{12}$ and iodine-oxidized $Li_{3+\delta}Fe_3Te_2O_{12}$.

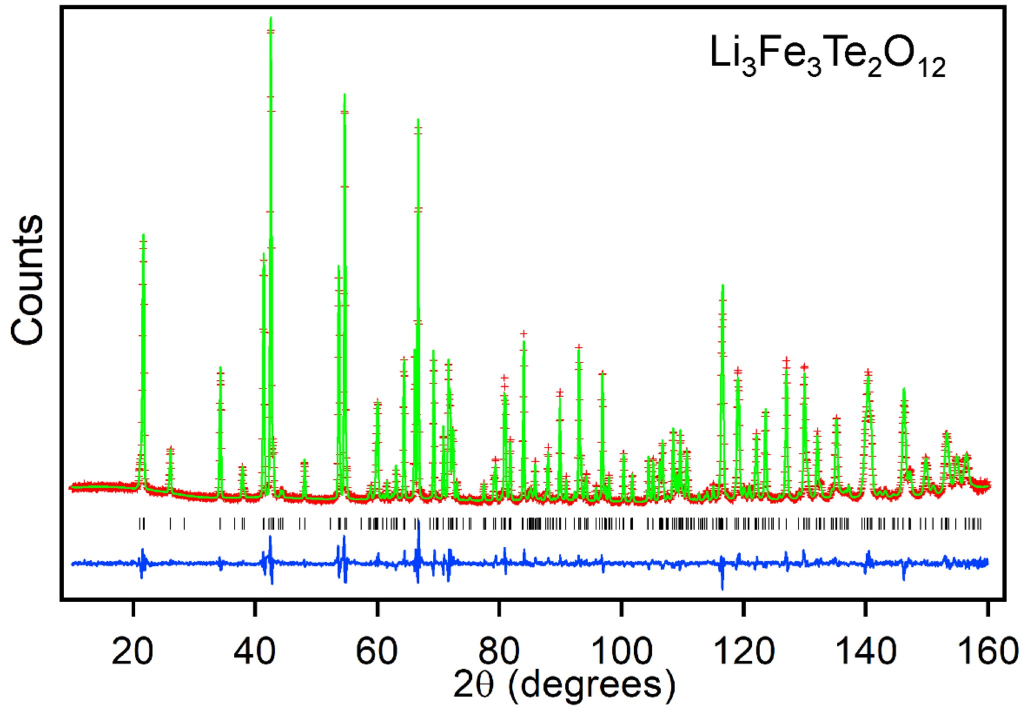

Figure S1. Observed, calculated and difference plots from the structural refinement of $Li_3Fe_3Te_2O_{12}$ against NPD data collected at room temperature.

Atom	Site	*x*	*y*	*z*	Occupancy	B_{eq} (Å^2)
Te	2*a*	0	0	0	1	0.014(3)
Fe/Li	4*f*	0	½	0.8021(1)	0.75/0.25	0.14(2)
Li	8*h*	0.041(4)	0.045(3)	0.436(2)	0.25	2.9(3)
O1	8*h*	0.2770(2)	0.3365(2)	0.3383(1)	1	0.41(1)
O2	4*g*	0.6986(3)	0.1819(3)	½	1	0.51(2)
$Li_3Fe_3Te_2O_{12}$ – space group *Pnnm (#58)*<br>*a* = 4.91156(3) Å, *b* = 5.08418(3) Å, *c* = 8.42437(7) Å, *volume* = 210.367(3) Å^3<br>Formula weight = 635.57 g mol^{-1},<br>Radiation source: Constant wavelength neutron, λ = 1.594 Å<br>Temperature: 298 K<br>R_p = 4.09 %, $_wR_p$ = 5.41 %						

Table S1. Parameters from the structural refinement of $Li_3Fe_3Te_2O_{12}$ against NPD data collected at room temperature.

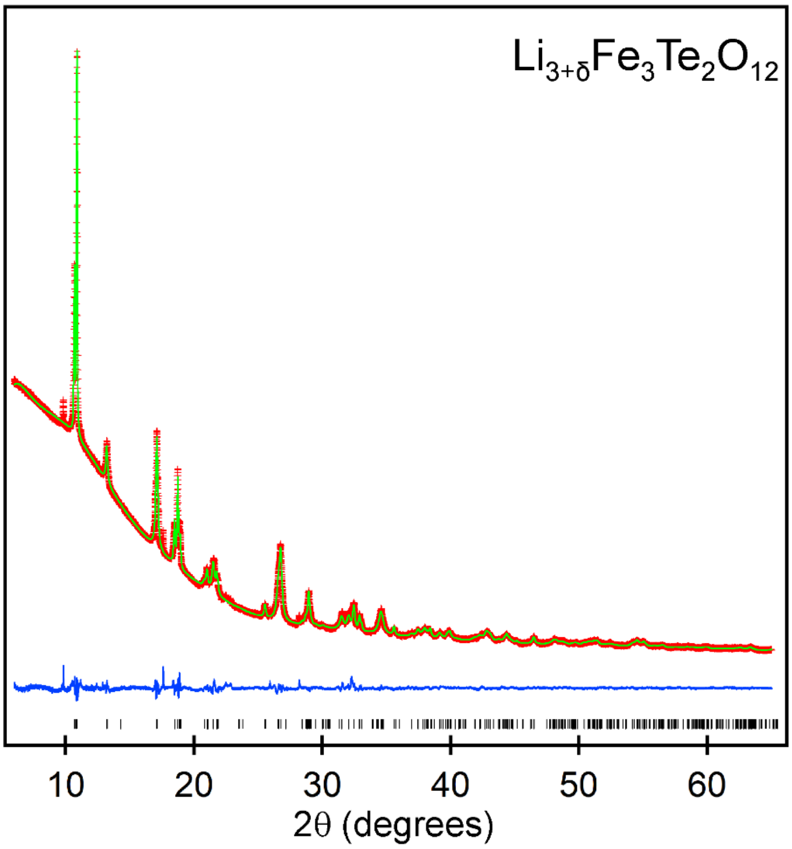

Figure S2. Observed, calculated and difference plots from the structural refinement of $Li_{3+\delta}Fe_3Te_2O_{12}$ against SXRD data collected at room temperature.

Atom	Site	*x*	*y*	*z*	Occupancy	B_{eq} (Å^2)
Te	2*a*	0	0	0	1	0.56(2)
Fe/Li	4*f*	0	½	0.8077(2)	0.555(3)/0.445(3)	0.74(5)
Li/Fe	4*e*	0	0	0.6527(5)	0.5+x/0.194(3)	0.74(5)
O1	8*h*	0.2879(9)	0.3585(7)	0.3343(6)	1	0.62(7)
O2	4*g*	0.747(1)	0.228(1)	½	1	0.62(7)

$Li_{3+\delta}Fe_3Te_2O_{12}$ – space group *Pnnm* (#58)

a = 5.0263(1) Å, *b* = 5.1480(1) Å, *c* = 8.7616(1) Å, *volume* = 226.71(1) Å^3

Formula weight = 635.57 + δ×6.94 g mol^{-1},

Radiation source: Synchrotron X-ray, λ = 0.826860 Å

Temperature: 298 K

R_p = 1.05 %, $_wR_p$ = 1.48 %

Table S2. Parameters from the structural refinement of $Li_{3+\delta}Fe_3Te_2O_{12}$ against SXRD data collected at room temperature.

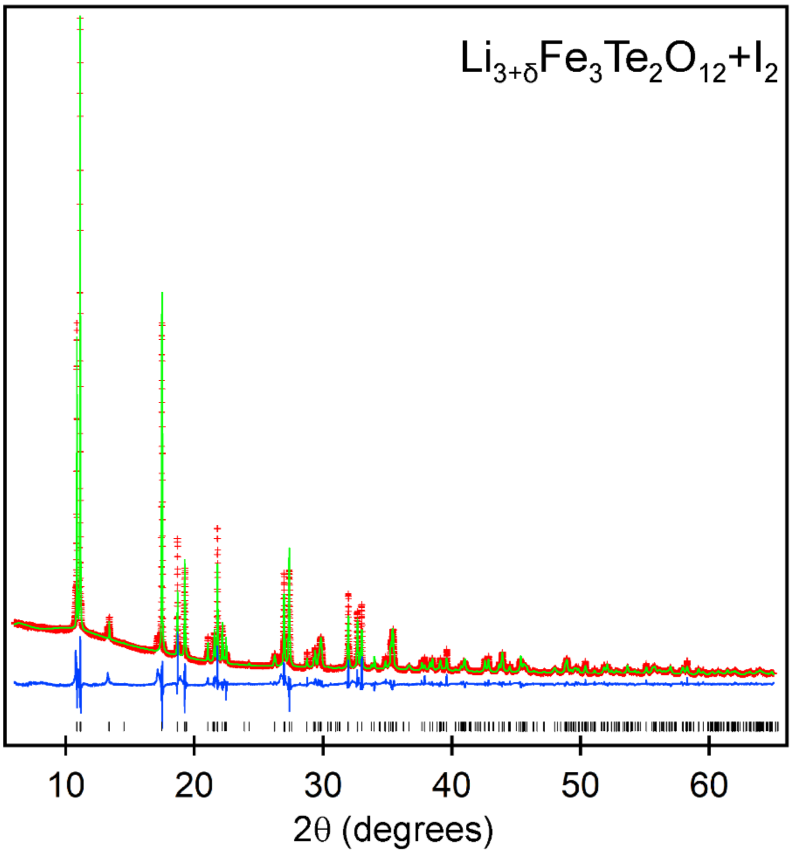

Figure S3. Observed, calculated and difference plots from the structural refinement of $Li_{3+\delta}Fe_3Te_2O_{12}$ which had been reacted with iodine, against SXRD data collected at room temperature.

Atom	Site	*x*	*y*	*z*	Occupancy	B_{eq} (Å^2)
Te	2*a*	0	0	0	1	0.64(2)
Fe/Li	4*f*	0	½	0.8021(1)	0.75(1)/0.25(1)	0.94(1)
Li	8*h*	0.041	0.045	0.436	0.25	0.94(1)
O1	8*h*	0.282(1)	0.341(1)	0.335(1)	1	0.73(1)
O2	4*g*	0.701(1)	0.187(1)	½	1	0.73(1)
$Li_3Fe_3Te_2O_{12}$ – space group *Pnnm* (#58) (I_2 treated)<br>*a* = 4.9418(1) Å, *b* = 5.0963(1) Å, *c* = 8.4920(2) Å, *volume* = 213.87(1) Å^3<br>Formula weight = 635.57 g mol^{-1},<br>Radiation source: Synchrotron X-ray, λ = 0.826860 Å<br>Temperature: 298 K<br>R_p = 4.48 %, $_wR_p$ = 7.29 %						

Table S3. Parameters from the structural refinement of $Li_{3+\delta}Fe_3Te_2O_{12}$, which had been treated with I_2, against SXRD data collected at room temperature. The position of the lithium 8*h* position was not refined due to the low X-ray scattering powder of lithium.

	$Li_3Fe_3Te_2O_{12}$	$Li_{3+\delta}Fe_3Te_2O_{12}$	$Li_{3+\delta}Fe_3Te_2O_{12}$ + I_2
Te 2*a* site			
O(1) × 4	1.935(1)	1.951(5)	1.944(8)
O(2) × 2	1.889(2)	1.871(7)	1.879(8)
<Te-O>	1.919	1.924	1.922
Fe/Li 4*f*			
O1× 2	1.985(1)	2.033(5)	1.989(8)
O1× 2	2.054(1)	2.151(4)	2.064(6)
O2× 2	2.141(1)	2.403(5)	2.172(5)
<Fe-O>	2.060	2.165	2.074
BVS	Fe+2.69	Fe+1.99	Fe+2.60
Fe:Li	75:25	55:45	75(1):25(1)
Li/Fe 4*e*			
O1× 2		2.057(7)	
O1× 2		2.339(4)	
O2× 2		2.187(6)	
<M-O>		2.194	
BVS (Fe)		Fe+1.94	
Fe:Li		0.19:Li	

Table S4. Selected bond lengths (Å) from the refined structures of $Li_3Fe_3Te_2O_{12}$, $Li_{3+\delta}Fe_3Te_2O_{12}$ and iodine-treated $Li_{3+\delta}Fe_3Te_2O_{12}$.

2. Structural characterisation of $Li_3Fe_2InTe_2O_{12}$, $Li_{3+\delta}Fe_2InTe_2O_{12}$ and iodine-oxidized $Li_{3+\delta}Fe_2InTe_2O_{12}$.

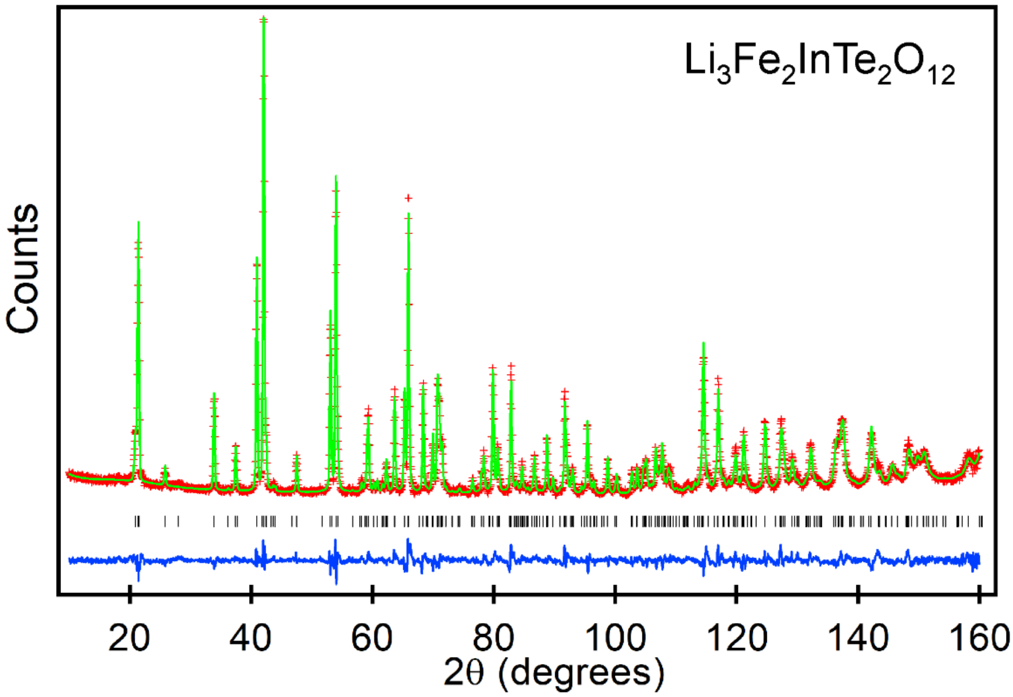

Figure S4. Observed, calculated and difference plots from the structural refinement of $Li_3Fe_3Te_2O_{12}$ against NPD data collected at room temperature.

Atom	Site	*x*	*y*	*z*	Occupancy	B_{eq} (Å^2)
Te	2*a*	0	0	0	1	0.08(2)
Fe/In/Li	4*f*	0	½	0.7996(2)	0.50/25/0.25	0.36(4)
Li	8*h*	0.031(6)	0.044(5)	0.442(2)	0.25	2.8(3)
O1	8*h*	0.2807(4)	0.3388(3)	0.3401(2)	1	0.69(3)
O2	4*g*	0.7019(6)	0.1877(4)	½	1	0.44(4)
$Li_3Fe_2InTe_2O_{12}$ – space group *Pnnm* (#58)<br>*a* = 4.97057(8) Å, *b* = 5.13561(8) Å, *c* = 8.5224(1) Å, *volume* = 217.552(7) Å^3<br>Formula weight = 694.54 g mol^{-1},<br>Radiation source: Constant wavelength neutron, λ = 1.594 Å<br>Temperature: 298 K<br>R_p = 4.53 %, $_wR_p$ = 5.48 %						

Table S5. Parameters from the structural refinement of $Li_3Fe_2InTe_2O_{12}$ against NPD data collected at room temperature.

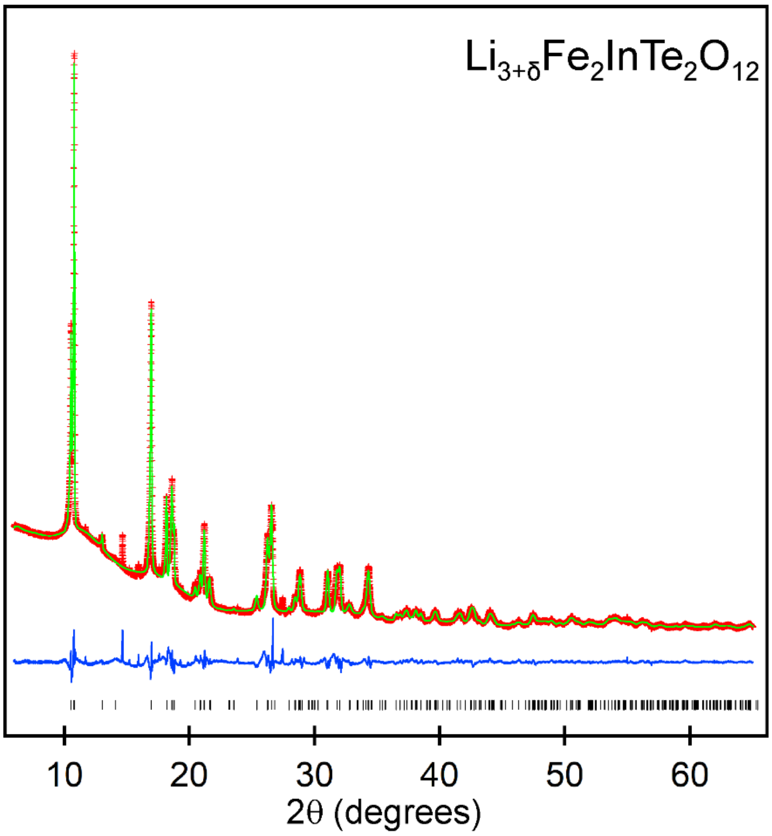

Figure S5. Observed, calculated and difference plots from the structural refinement of $Li_{3+\delta}Fe_2InTe_2O_{12}$ against SXRD data collected at room temperature.

Atom	Site	*x*	*y*	*z*	Occupancy	B_{eq} (Å^2)
Te	2*a*	0	0	0	1	1.33(4)
Fe/In/Li	4*f*	0	½	0.8024(1)	0.404(1)/0.25/0.345(1)	1.61(6)
Li/Fe	4*e*	0	0	0.669(1)	0.25+x/0.095(1)	1.61(6)
O1	8*h*	0.3033(8)	0.3439(7)	0.3383(8)	1	0.77(7)
O2	4*g*	0.719(1)	0.201(1)	½	1	0.77(7)
$Li_{3+x}Fe_2InTe_2O_{12}$ – space group *Pnnm* (#58)						
a = 5.0622(2) Å, *b* = 5.2216(1) Å, *c* = 8.7732(3) Å, *volume* = 231.90(1) Å^3						
Formula weight = 694.54 + δ×6.94 g mol^{-1},						
Radiation source: Synchrotron X-ray, λ = 0.826860 Å						
Temperature: 298 K						
R_p = 1.41 %, $_wR_p$ = 2.16 %						

Table S6. Parameters from the structural refinement of $Li_{3+\delta}Fe_2InTe_2O_{12}$ against SXRD data collected at room temperature.

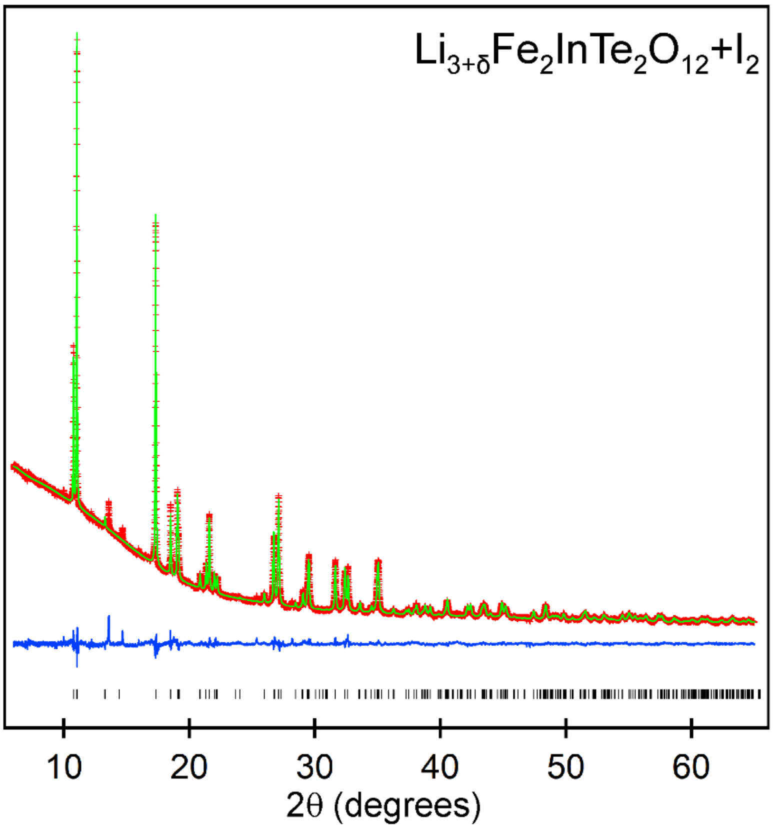

Figure S6. Observed, calculated and difference plots from the structural refinement of $Li_{3+\delta}Fe_2InTe_2O_{12}$ which had been reacted with iodine, against SXRD data collected at room temperature.

Atom	Site	*x*	*y*	*z*	Occupancy	B_{eq} (Å^2)
Te	2*a*	0	0	0	1	0.31(2)
Fe/In/Li	4*f*	0	½	0.7985(1)	0.412(1)/0.25/0.338(1)	0.59(3)
Li/Fe	4*e*	0	0	0.609(1)	0.25+x/0.088(1)	0.59(3)
O1	8*h*	0.278(1)	0.3406(6)	0.3396(6)	1	0.28(6)
O2	4*g*	0.713(1)	0.182(1)	½	1	0.28(6)
$Li_{3+\delta}Fe_2InTe_2O_{12}$ – space group *Pnnm* (#58) (I_2 treated)<br>*a* = 4.9810(1) Å, *b* = 5.1484(1) Å, *c* = 8.5867(1) Å, *volume* = 220.207(8) Å^3<br>Formula weight = 694.54 + δ×6.94 g mol^{-1},<br>Radiation source: Synchrotron X-ray, λ = 0.826860 Å<br>Temperature: 298 K<br>R_p = 1.41 %, $_wR_p$ = 2.16 %						

Table S7. Parameters from the structural refinement of $Li_{3+\delta}Fe_2InTe_2O_{12}$, which had been treated with I_2, against SXRD data collected at room temperature.

	$Li_3Fe_2InTe_2O_{12}$	$Li_{3+\delta}Fe_2InTe_2O_{12}$	$Li_{3+\delta}Fe_2InTe_2O_{12}$ + I_2
Te 2*a* site			
O(1) × 4	1.931(1)	1.915(6)	1.949(6)
O(2) × 2	1.892(3)	1.914(8)	1.951(6)
<Te-O>	1.918	1.914	1.947
Fe/In/Li 4*f*			
O1× 2	2.012(3)	2.077(4)	2.002(6)
O1× 2	2.082(3)	2.132(6)	2.102(5)
O2× 2	2.203(3)	2.313(6)	2.238(5)
<Fe-O>	2.099	2.174	2.113
BVS	Fe+2.44	Fe+2.02	Fe+2.37
Fe:In:Li	0.5:0.25:0.25	0.404(1):0.25:0.345(1)	0.412(1):0.25:0.338(1)
Li/Fe 4*e*			
O1× 2		1.961(11)	2.279(6)
O1× 2		2.364(4)	2.408(10)
O2× 2		2.310(10)	1.949(7)
<M-O>		2.211	2.211
BVS (Fe)		Fe+1.999	Fe+2.033
Fe:Li		0.09(1):0.25+x	0.088(1) :0.25+x

Table S8. Selected bond lengths (Å) from the refined structures of $Li_3Fe_2InTe_2O_{12}$, $Li_{3+\delta}Fe_2InTe_2O_{12}$ and iodine-treated $Li_{3+\delta}Fe_2InTe_2O_{12}$.

3. Structural characterisation of $Li_3Fe_{2.6}Al_{0.4}Te_2O_{12}$, $Li_{3+\delta}Fe_{2.6}Al_{0.4}Te_2O_{12}$ and iodine-oxidized $Li_{3+\delta}Fe_{2.6}Al_{0.4}Te_2O_{12}$.

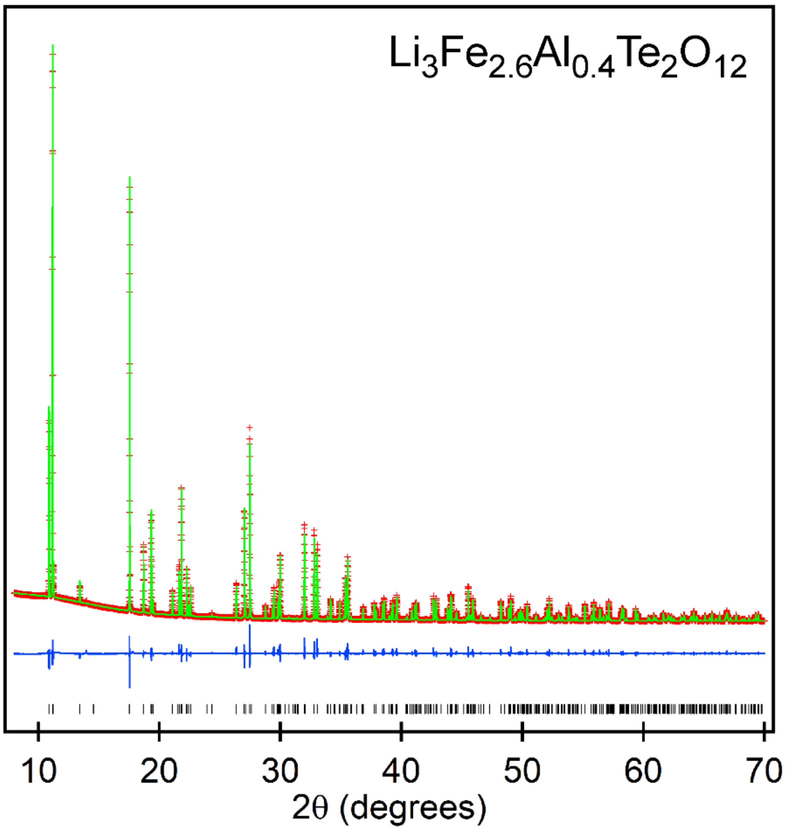

Figure S7. Observed, calculated and difference plots from the structural refinement of $Li_3Fe_{2.6}Al_{0.4}Te_2O_{12}$ against SXRD data collected at room temperature.

Atom	Site	*x*	*y*	*z*	Occupancy	B_{eq} (Å^2)
Te	2*a*	0	0	0	1	0.12(2)
Fe/Al/Li	4*f*	0	½	0.8014(1)	0.65/0.1/0.25	0.39(4)
Li	8*h*	0.031	0.044	0.442	0.25	1.0
O1	8*h*	0.2757(3)	0.3354(2)	0.3373(1)	1	0.67(2)
O2	4*g*	0.6958(3)	0.1772(3)	½	1	0.67(2)
$Li_3Fe_{2.4}Al_{0.6}Te_2O_{12}$ – space group *Pnnm* (#58)<br>*a* = 4.92291(1) Å, *b* = 5.09674(1) Å, *c* = 8.45182(1) Å, *volume* = 212.063(1) Å^3<br>Formula weight = 624.02 g mol^{-1},<br>Radiation source: Synchrotron X-ray, λ = 0.826860 Å<br>Temperature: 298 K<br>R_p = 2.01 %, $_wR_p$ = 3.34 %						

Table S9. Parameters from the structural refinement of $Li_3Fe_{2.6}Al_{0.4}Te_2O_{12}$ against SXRD data collected at room temperature.

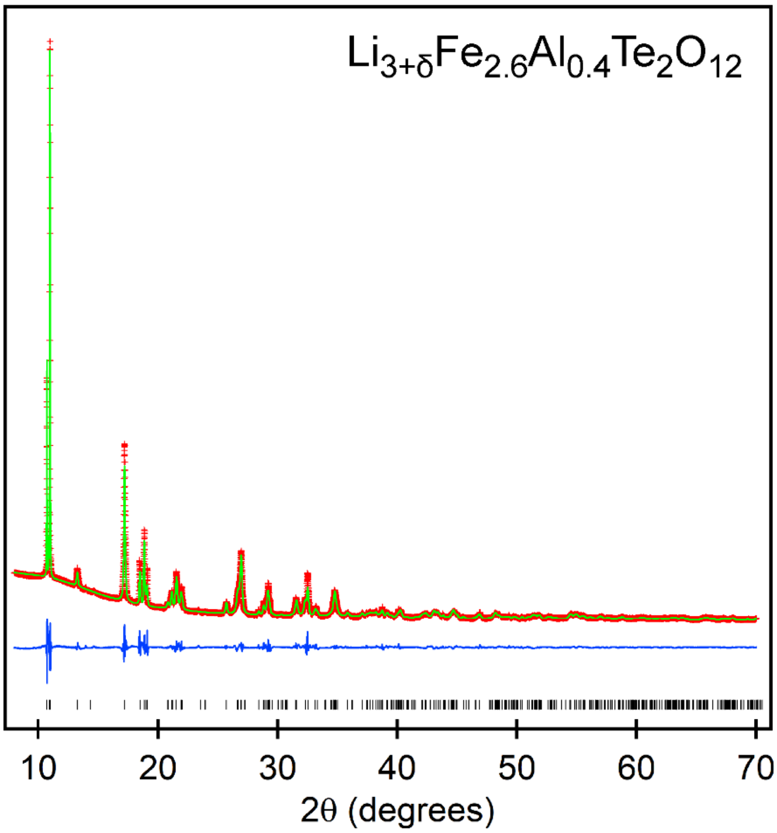

Figure S8. Observed, calculated and difference plots from the structural refinement of $Li_{3+\delta}Fe_{2.6}Al_{0.4}Te_2O_{12}$ against SXRD data collected at room temperature.

Atom	Site	*x*	*y*	*z*	Occupancy	B_{eq} (Å^2)
Te	2*a*	0	0	0	1	0.52(2)
Fe/Al/Li	4*f*	0	½	0.8058(1)	0.608(1)/0.1/0.291(1)	0.64(3)
Li/Fe	4*e*	0	0	0.644(1)	0.958(1)/0.041(1)	0.64(3)
O1	8*h*	0.278(1)	0.3442(7)	0.3410(6)	1	0.83(6)
O2	4*g*	0.734(1)	0.183(1)	½	1	0.83(6)
$Li_{3+\delta}Fe_{2.6}Al_{0.4}Te_2O_{12}$ – space group *Pnnm* (#58)<br>*a* = 4.9879(1) Å, *b* = 5.1488(1) Å, *c* = 8.6982(1) Å, *volume* = 223.38(1) Å^3<br>Formula weight = 624.02 + δ×6.94 g mol^{-1},<br>Radiation source: Synchrotron X-ray, λ = 0.826860 Å<br>Temperature: 298 K<br>R_p = 2.51 %, $_wR_p$ = 4.19 %						

Table S10. Parameters from the structural refinement of $Li_{3+\delta}Fe_{2.6}Al_{0.4}Te_2O_{12}$ against SXRD data collected at room temperature.

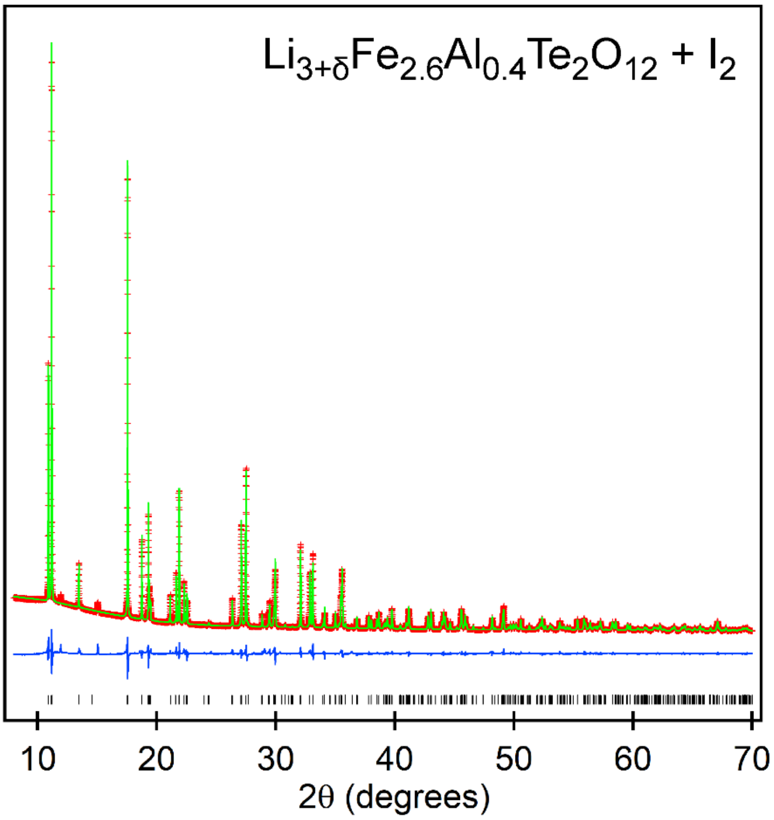

Figure S9. Observed, calculated and difference plots from the structural refinement of $Li_{3+\delta}Fe_{2.6}Al_{0.4}Te_2O_{12}$ which had been reacted with iodine, against SXRD data collected at room temperature.

Atom	Site	*x*	*y*	*z*	Occupancy	B_{eq} (Å^2)
Te	2*a*	0	0	0	1	0.21(1)
Fe/Al/Li	4*f*	0	½	0.8009(1)	0.632(1)/0.1/0.267(1)	0.94(3)
Li/Fe	4*e*	0	0	0.6199(1)	00.983(1)/0.017(1)	0.94(3)
O1	8*h*	0.2795(4)	0.3383(3)	0.3357(2)	1	0.35(2)
O2	4*g*	0.7068(5)	0.1837(5)	½	1	0.35(2)
$Li_{3+\delta}Fe_{2.6}Al_{0.4}Te_2O_{12}$ – space group *Pnnm* (#58)<br>*a* = 4.9108(1) Å, *b* = 5.0787(1) Å, *c* = 8.4633(1) Å, *volume* = 211.08(1) Å^3<br>Formula weight = 624.02 + δ×6.94 g mol^{-1},<br>Radiation source: Synchrotron X-ray, λ = 0.826860 Å<br>Temperature: 298 K<br>R_p = 2.56 %, $_wR_p$ = 3.92 %						

Table S11. Parameters from the structural refinement of $Li_{3+\delta}Fe_{2.6}Al_{0.4}Te_2O_{12}$, which had been treated with I_2, against SXRD data collected at room temperature.

	$Li_3Fe_{2.6}Al_{0.4}Te_2O_{12}$	$Li_{3+\delta}Fe_{2.6}Al_{0.4}Te_2O_{12}$	$Li_{3+\delta}Fe_{2.6}Al_{0.4}Te_2O_{12}$ + I_2
Te 2*a* site			
O(1) × 4	1.953(1)	1.944(6)	1.944(1)
O(2) × 2	1.907(3)	2.055(7)	1.900(3)
<Te-O>	1.937	1.964	1.929
Fe/Al/Li 4*f*			
O1× 2	1.980(1)	2.049(6)	1.974(1)
O1× 2	2.057(1)	2.112(5)	2.052(1)
O2× 2	2.136(1)	2.262(5)	2.177(1)
<Fe-O>	2.058	2.141	2.068
BVS	Fe+2.72	Fe+2.20	Fe+2.67
Fe:Al:Li	0.65:0.1:0.25	0.609(1):0.1:0.291(1)	0.632(1):0.1:0.268(1)
Li/Fe 4*e*			
O1× 2		2.186(1)	2.231(2)
O1× 2		2.255(5)	2.276(5))
O2× 2		2.058(8)	1.994(4)
<M-O>		2.166	2.167
BVS (Fe)		Fe+2.05	Fe+2.11
Fe:Li		0.041(1):0.958(1)	0.018(1):0.982(1)

Table S12. Selected bond lengths (Å) from the refined structures of $Li_3Fe_2InTe_2O_{12}$, $Li_{3+\delta}Fe_2InTe_2O_{12}$ and iodine-treated $Li_{3+\delta}Fe_2InTe_2O_{12}$.

4. Chemical lithiation of $Li_3Fe_3Te_2O_{12}$ and $Li_3Fe_{2.6}Al_{0.4}Te_2O_{12}$.

As noted in the main text, exposure of $Li_3Fe_3Te_2O_{12}$ or $Li_3Fe_{2.6}Al_{0.4}Te_2O_{12}$ to *n*-BuLi for periods of more than 24 hours lead to the decomposition of the oxide phases. X-ray powder diffraction data collected from samples exposed to *n*-BuLi for more than 24 hours (see Figure S10 below) exhibit strong diffraction peaks from elemental Te, along with additional broad diffraction features which cannot be readily assigned to known phases, but which we attribute to other reduced reduction products.

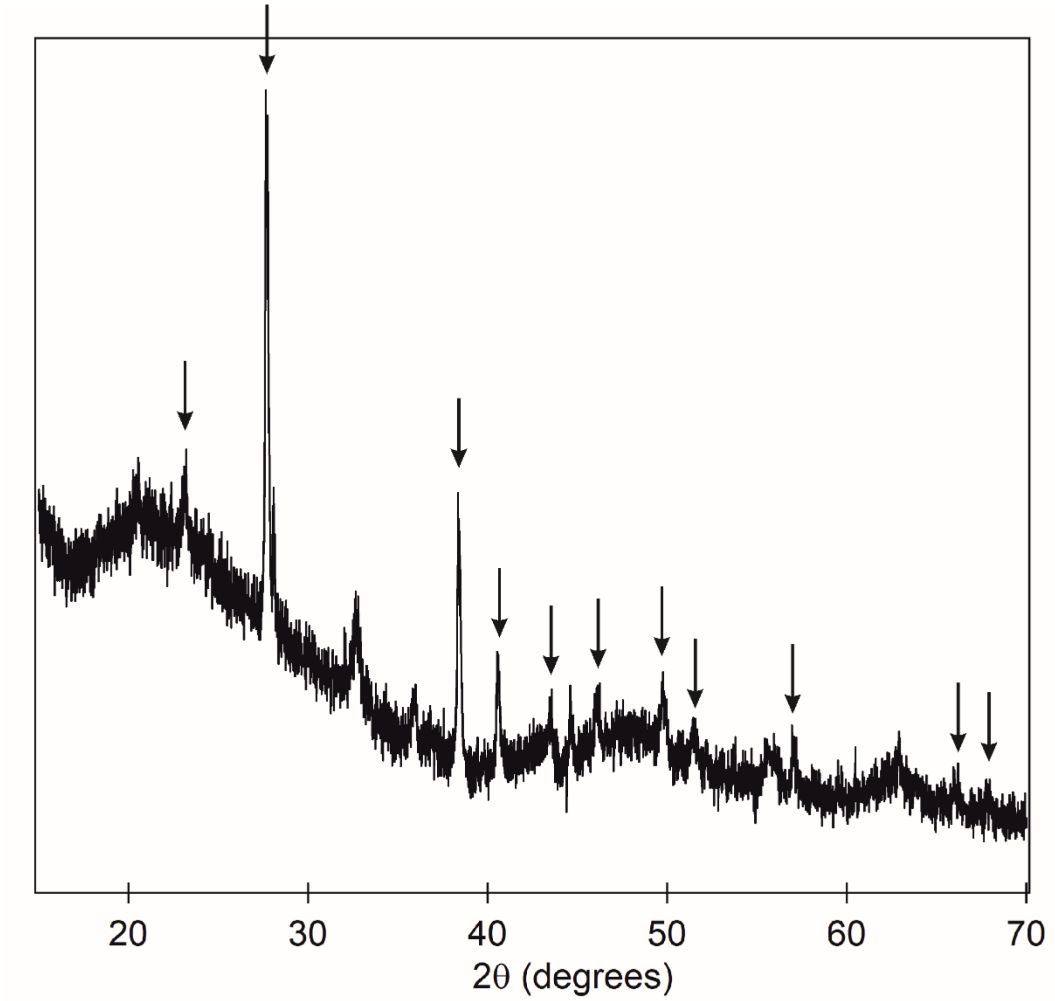

Figure S10. PXRD data collected from a sample of $Li_3Fe_3Te_2O_{12}$ exposed to *n*-BuLi for 10 days. Arrows indicate diffraction peaks due to elemental Te.
